# Sphingolipids as a Culprit of Mitochondrial Dysfunction in Insulin Resistance and Type 2 Diabetes

**DOI:** 10.3389/fendo.2021.635175

**Published:** 2021-03-18

**Authors:** Kamila Roszczyc-Owsiejczuk, Piotr Zabielski

**Affiliations:** ^1^Department of Medical Biology, Medical University of Bialystok, Bialystok, Poland; ^2^Department of Hygiene, Epidemiology and Metabolic Disorders, Medical University of Bialystok, Bialystok, Poland

**Keywords:** sphingolipids, insulin resistance, type 2 diabetes, mitochondrial dysfunction, metabolism, ceramide

## Abstract

Insulin resistance is defined as a complex pathological condition of abnormal cellular and metabolic response to insulin. Obesity and consumption of high-fat diet lead to ectopic accumulation of bioactive lipids in insulin-sensitive tissues. Intracellular lipid accumulation is regarded as one of the major factors in the induction of insulin resistance and type 2 diabetes (T2D). A significant number of studies have described the involvement of ceramides and other sphingolipids in the inhibition of insulin-signaling pathway in both skeletal muscles and the liver. Adverse effects of sphingolipid accumulation have recently been linked to the activation of protein kinase Cζ (PKCζ) and protein phosphatase 2A (PP2A), which, in turn, negatively affect phosphorylation of serine/threonine kinase Akt [also known as protein kinase B (PKB)], leading to decreased glucose uptake in skeletal muscles as well as increased gluconeogenesis and glycogenolysis in the liver. Sphingolipids, in addition to their direct impact on the insulin signaling pathway, may be responsible for other negative aspects of diabetes, namely mitochondrial dysfunction and deficiency. Mitochondrial health, which is characterized by appropriate mitochondrial quantity, oxidative capacity, controlled oxidative stress, undisturbed respiratory chain function, adenosine triphosphate (ATP) production and mitochondrial proliferation through fission and fusion, is impaired in the skeletal muscles and liver of T2D subjects. Recent findings suggest that impaired mitochondrial function may play a key role in the development of insulin resistance. Mitochondria stay in contact with the endoplasmic reticulum (ER), Golgi membranes and mitochondria-associated membranes (MAM) that are the main places of sphingolipid synthesis. Moreover, mitochondria are capable of synthesizing ceramide though ceramide synthase (CerS) activity. Recently, ceramides have been demonstrated to negatively affect mitochondrial respiratory chain function and fission/fusion activity, which is also a hallmark of T2D. Despite a significant correlation between sphingolipids, mitochondrial dysfunction, insulin resistance and T2D, this subject has not received much attention compared to the direct effect of sphingolipids on the insulin signaling pathway. In this review, we focus on the current state of scientific knowledge regarding the involvement of sphingolipids in the induction of insulin resistance by inhibiting mitochondrial function.

## Introduction

Overweight and obesity are serious, long-term conditions with a major impact on the development of other metabolic diseases, including cardiovascular disease, type 2 diabetes, and insulin resistance (1, 2). According to the World Health Organization, the prevalence of obesity in all age groups worldwide nearly tripled between the years 1975 and 2016. If the current trend continues, it is estimated that 177 million adults will be severely affected by obesity by 2025 (3). Moreover, it is predicted that the worldwide prevalence of diabetes among adults will increase from 6.4% in 2010 to 7.7% by 2030 (4). Sphingolipids (SL) represent a major class of lipids that are ubiquitous components of eukaryotic cells in which they play an important role as building blocks of biological membranes. Furthermore, they control critical cellular functions, such as the cell cycle, senescence, apoptosis, cell migration and inflammation (5). Multiple studies conducted in the past decades revealed that members of the SL family, including ceramide (Cer), sphingosine (SPH), sphingosine-1 phosphate (S1P) and ceramide-1-phosphate (C1P) act as bioactive molecules which control numerous signal transduction pathways (6). Sphingolipids are synthesized *via* a complex metabolic pathway and their intracellular levels are tightly regulated by various enzymatic processes (7). Sphingolipids, in addition to their direct impact on the molecular pathways, may modulate mitochondrial function, adversely affecting cellular energy and redox metabolism – one of the hallmarks of T2D. Increased content of intracellular ceramide impairs mitochondrial function by interfering with various aspects of mitochondrial electron transport chain (ETC), mitochondrial respiration, oxidative phosphorylation (OXPHOS) and ATP production, mitochondrial biogenesis and fission-fusion dynamics.

Mitochondria are highly dynamic organelles responsible for fulfilling cellular energy requirements through ATP production. A mitochondrion is structurally divided into four sections: outer mitochondrial membrane (OMM), the intermembrane space, inner mitochondrial membrane (IMM) and matrix where tricarboxylic acid (TCA) cycle and β-oxidation of fatty acids (FA) take place. It has been reported that mitochondrial membranes contain a variety of ceramide species differing in acyl chain length and saturation. However, their detailed composition and origin are currently not well understood (8). The inner mitochondrial membrane enables the transport of otherwise impermeable adenosine diphosphate (ADP), phosphate and ATP, and anchors multi-subunit complexes of electron transport chain proteins (9). The mitochondrial ETC is composed of five multi-subunit enzyme complexes I, II, III, IV and V located in the inner mitochondrial membrane. Electrons from NADH and FADH_2_ enter the electron transport chain through complex I and complex II. Afterwards, electrons are transported to complex III through coenzyme Q and then to complex IV through cytochrome c (Cyt c). The released energy is transformed into the electrochemical proton gradient across the inner mitochondrial membrane which acts as the driving force for ATP synthesis *via* complex V activity (ATP synthase) (10, 11). In addition to energy production, mitochondria are the source of reactive oxygen species (ROS) which – if not strictly controlled – lead to oxidative damage to proteins, lipids and mitochondrial DNA (12). Recent findings suggest that nutrient oversupply and subsequent obesity negatively affect the activity of the mitochondrial electron transport chain and oxidative phosphorylation, as well as boost ROS production and mitochondrial fragmentation. It has been demonstrated that obesity, T2D and insulin resistance are connected with at least one of the aspects of mitochondrial dysfunction in a tissue-dependent manner (13). The definition of mitochondrial dysfunction can be determined based on numerous functions that mitochondria perform in cells. Obesity, insulin resistance and T2D diminish the oxidative capacity of mitochondria (14) and their ability to produce ATP (15), increase oxidative stress (16), alter mitochondrial network dynamics through the fission-fusion process (17), disrupt mitophagy (18), decrease mitochondrial DNA (mtDNA) copy number (19) and affect mitochondrial morphology and content (20). Recent findings suggest that sphingolipid-driven defects in mitochondrial metabolic fitness and network dynamics may play a key role in the development of obesity, insulin resistance and T2D. This review is focused on the sphingolipid-mitochondria interaction through critical steps of sphingolipid synthesis in the ER, transport of ceramide from the ER to the mitochondria, distinct mitochondrial pathways of ceramide metabolism and various consequences of sphingolipid accumulation in the mitochondria, highlighting the role of sphingolipids in the regulation of mitochondrial function in obesity-induced insulin resistance.

## Ceramide and Insulin Resistance – Direct Inhibition of Insulin Signaling Pathway

One of the features of obesity is the accumulation of ectopic fat in non-adipose tissues, which is believed to induce insulin resistance (21). Due to the joint occurrence of insulin resistance and ectopic fat accumulation, the majority of studies focus on the immediate effect of lipids on the constituents of the insulin signaling pathway. Insulin binds to the extracellular α subunit of the insulin receptor (IR), thus triggering the phosphorylation of the receptor β subunit harboring tyrosine kinase activity. Auto-phosphorylation of the receptor activates a signaling cascade and enables tyrosine phosphorylation of intracellular substrate protein known as insulin receptor substrate 1 (IRS-1) (22–24). IRS-1 was first cloned and described by Morris F. White’s team in 1991 as an important adaptor protein which relays a phosphorylation signal from IR to other molecular targets (25). The subsequent signaling cascade leads to the activation of phosphatidylinositol 3-kinase (PI3K), conversion of membrane-bound phosphatidylinositol 4,5-bisphosphate (PIP2) to phosphatidylinositol (3,4,5)-trisphosphate (PIP3) and activation of protein kinase B (PKB, also known as Akt protein). The effect of insulin on the skeletal muscle and adipose tissue glucose uptake *via* Akt/PKB activation is due to its ability to stimulate the translocation of glucose transporter type-4 (GLUT4) -containing vesicles from the cytoplasm to the cell membrane through AS160 adapter protein phosphorylation. In addition to direct effect on glucose uptake, insulin – through the PI3K-regulated pathway – influences the activity of a number of kinases that control glycolysis, lipid synthesis, protein synthesis and glycogenesis (26–28). Ceramides can directly inhibit insulin signaling through two major mechanisms. Chavez et al. showed that exposing muscle cells to particular saturated free fatty acids leads to ceramide accumulation and hinders insulin-stimulated Akt/PKB phosphorylation. Those effects could be reversed by the inhibitors of ceramide synthesis, i.e., myriocin and fumonisin B1 (29). Ceramides activate protein phosphatase A2 (PP2A) which dephosphorylates Akt/PKB at T308 moiety (30, 31). Furthermore, they block the translocation of serine/threonine kinase Akt/PKB to the plasma membrane *via* a mechanism based on atypical protein kinase Cζ (PKCζ). Hajduch et al. demonstrated that ceramide-induced Akt/PKB inhibition is critically dependent on the targeting and subsequent retention of PKCζ within CEM (caveolin-enriched microdomains) in adipocytes and skeletal muscles (32–34). Both mechanisms are currently well examined and believed to be responsible for immediate effects of lipid oversupply on tissue insulin sensitivity. Indirect consequences of ceramide accumulation arise from its impact on mitochondria which are situated close to the origin of sphingolipid biosynthesis and remain in close contact with ER through mitochondria-associated membranes and unique transport mechanisms.

## Mitochondrial Sphingolipids and Their Origin

Using LC-MS profiling, Bird et al. identified 22 unique ceramide, 6 sphingomyelin (SM), and 4 ganglioside (GM) species in rat liver mitochondria (35). A number of enzymes in the sphingolipid biosynthesis pathway have been detected in the mitochondria, including ceramide synthase, ceramidase (CDase), sphingomyelinase (SMase) and sphingosine kinase (SphK) (36–38). The research conducted by Shimeno et al. confirmed the presence of ceramide synthase in the liver mitochondria which drives mitochondrial ceramide production through N-acylation of sphinganine (from *de novo* pathway) and sphingosine derived from the mitochondrial salvage pathway (36). The presence of ceramide synthase was observed not only in whole mitochondria but also in mitochondrial membrane compartments. CerS activity was recovered from both the outer and inner mitochondrial membrane as well from MAM purified from rat liver (39). Birbes et al. confirmed the presence of mitochondrial neutral sphingomyelinase (nSMase) in the outer mitochondrial membrane (40), whereas Novgorodov et al. identified the presence of nCDase and sphingosine kinase 2 (SphK2) in mouse brain mitochondria. Both enzymes co-precipitate with mitochondrial cytochrome oxidase (COX) subunit 1 and play a major role in the mitochondrial SPH/S1P rheostat in ischemic brain injury (37). SphK2 (which has been demonstrated to be the major source of mitochondrial S1P) interacts with prohibitin 2 (PHB2), a highly conserved protein that regulates mitochondrial assembly and function. Moreover, mitochondrial SphK2 and S1P may regulate mitochondrial dynamics, the activity of the electron transport chain and mitochondrial respiration (38). Collectively, the above data clearly indicate that mitochondria and MAM have partially independent sphingolipid metabolism with sphingoid backbones supplied through the sphingosine salvage pathway. Furthermore, the presence of sphingolipids in both the IMMs and MOMs may point to a direct interaction of various sphingolipid molecular species with respiratory-chain components and other membrane proteins and, consequently, induce mitochondrial dysfunction.

**Figure 1:**
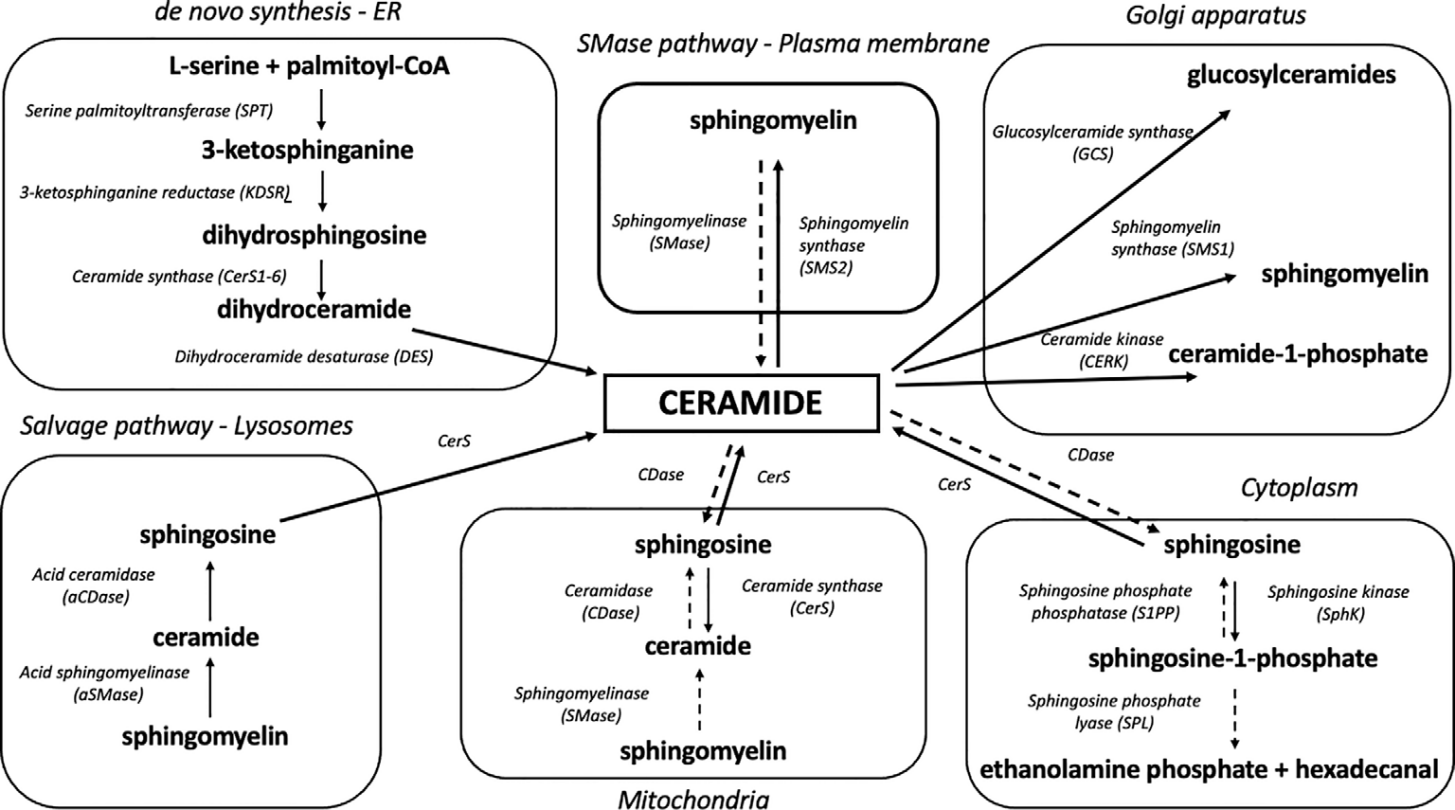
Organelle-specific pathways of sphingolipid metabolism. Due to its highly hydrophobic nature, ceramide is confined to the place of its release at the level of biological membranes. Ceramide degradation yields sphingosine which can shift between intracellular compartments and participate in sphingolipid synthesis in various organelles. Solid arrows denote synthesis reactions, whereas dotted arrows indicate the degradation of a given sphingolipid.

Eukaryotic cells are characterized by a complex internal structure organized into membrane-bound compartments (i.e., nuclear envelope, endoplasmic reticulum, Golgi apparatus, mitochondria, endosome, lysosome, peroxisome and lipid droplets) (41). These organelles interact with each other through vesicular transport and membrane contact sites (MCSs) which are essential to organelle biogenesis, growth and exchange of lipids and proteins (42). The endoplasmic reticulum is the primary area of sphingolipid biosynthesis in mammalian cells (43) and the major place of *de novo* synthesis of ceramide (**Figure 1**). Ceramide production involves at least three distinctive pathways located at different cellular compartments. It is synthesized mostly through a *de novo* pathway at the cytoplasmic leaflet of the ER and translocated to the mitochondria *via* mitochondria-associated membranes as part of lipid exchange between these two compartments. Other pathways of mitochondrial ceramide formation include: the recycling (salvage) pathway which liberates the sphingoid backbone through ceramide degradation at the lysosome level, followed by resynthesis in the mitochondria and hydrolysis of complex sphingolipids such as SM, which takes place in the Golgi and plasma membranes and has recently been identified also in the mitochondria (44–46).

### *De Novo* Biosynthetic Pathway

The *de novo* biosynthetic pathway occurs at the level of the endoplasmic reticulum and is governed by four enzymes: serine palmitoyl transferase (SPT), 3-ketodihydrosphingosine reductase (KDSR), ceramide synthases (CerS, various isoforms) and dihydroceramide desaturase (DES) (47). As a result of l-serine and palmitoyl-CoA condensation performed by SPT, 3-ketosphinganine (3-KSph) is formed and then subsequently reduced to dihydrosphingosine (dhSph, also described as sphinganine, Sph) by 3-ketosphinganine reductase (3-KR). This NADPH-dependent reaction produces a fundamental portion of sphingolipid metabolism – the sphingoid backbone. Dihydrosphingosine is then acylated with fatty acyl-CoA by ceramide synthase isoforms to dihydroceramide (dhCer). The final stage of the pathway ends with dihydroceramide desaturation catalyzed by the enzyme dihydroceramide desaturase (DES) which utilizes NADH or NADPH to produce a double bond in the C4-C5 position of the dihydrosphingosine backbone in order to yield ceramide (48) (**Figure 1**).

Up to date, six mammalian CerS have been identified (CerS1-6). Each of these enzymes has a unique expression profile as well as tissue distribution and display different affinity to fatty acid acyl-CoA chain length used for sphinganine N-acylation. Interestingly, although ceramide synthases have both common biochemical features and molecular structure, they affect insulin sensitivity and mitochondrial function differently. This suggests that individual molecular species of ceramide differ in their biological activity (49, 50). CerS1, the most abundant isoform of ceramide synthase in skeletal muscles, displays high affinity to stearoyl-CoA and is responsible for C18:0-Cer synthesis (51). Recent studies have underlined the importance of C18:0-Cer in the induction of skeletal muscle insulin resistance. Perreault et al. identified C18:0-Cer species as a key ceramide that negatively regulates insulin sensitivity in obese and/or T2D subjects (52). The lipidomic analysis of skeletal muscles obtained from insulin-resistant overweight/obese humans showed higher levels of C18:0-derived sphingolipids (53). Additionally, Bergman et al. demonstrated that C18:0 ceramide is uniquely related to insulin resistance in skeletal muscles during rest and exercise (54). Similarly, the studies conducted on Wistar rats (55) or mice (56) demonstrated that C18:0 and C18:1-derived ceramides play a leading role in fat-induced skeletal muscle insulin resistance. It has been demonstrated that the ablation of CerS1 expression in skeletal muscles may improve glucose tolerance under lipid overabundance. Turpin-Nolan et al. noted that increased CerS1 mRNA expression in the skeletal muscles of HFD-fed obese insulin resistant mice was accompanied by elevated skeletal muscle C18:0 ceramide content, whereas CerS1-deficient mice (CerS1ΔSkM) displayed decreased content of skeletal muscle C18:0-Cer and were protected from HFD-induced obesity  (57). The study by Turner et al. identified CerS1 as a potential endogenous inhibitor of mitochondrial fatty acid oxidation in skeletal muscles and a regulator of the whole-body adiposity (58). The use of isoform-specific CerS1 inhibitor (P053) in a HFD-fed mice enhanced skeletal muscle lipid oxidation through the augmentation of respiratory capacity. CerS1 inhibition significantly up-regulated mitochondria-encoded genes (Cox-2, Cytb, Atp6) and mitochondrial respiratory activity (through complex I, II and IV) and increased the activities of TCA cycle citrate synthase (CS) and β-oxidation marker enzyme β-hydroxyacyl CoA dehydrogenase (βHAD). According to one of the hypotheses formulated by the said authors, C18:0-Cer content reduced by CerS1 inhibition boosts respiratory capacity *via* the of mitochondrial fission (58).

CerS5 and CerS6 share an overlapping specificity for palmitoyl-CoA, yielding C16:0 ceramide (59). Recent studies have revealed that CerS6 may play an important role in the development of obesity and insulin resistance in the liver. C16:0-Cer reduction through CerS 5 or 6 silencing or inhibition could be a potential target for the treatment of insulin resistance and type 2 diabetes (60). The study conducted by Gosejacob et al. demonstrated that CerS5 is essential to maintain C16:0 sphingolipid pools in plasma, lung, spleen, muscle, liver, and white adipose tissue. CerS5 KO mice show improved indices of insulin resistance, reduced adiposity and decreased white adipose tissue inflammation after high fat diet challenge (61). Turpin et al. demonstrated a strong positive correlation between BMI value and C16:0 ceramide in the visceral and subcutaneous WAT of obese humans as well as in WAT and liver of HFD-fed mice (62). Moreover, the study revealed that CerS6Δ/Δ mice were protected from diet-induced obesity and had improved whole-body glucose metabolism and hepatic insulin signaling (62). This is in line with other findings that demonstrated a positive correlation between C16:0 ceramide content in the subcutaneous fat tissue and HOMA-IR (63). The link between CerS6, C16:0-Cer and mitochondrial function in the context of insulin resistance and obesity was observed in the mice model of liver-specific CerS6 ablation (CerS6Δ/Δ). The study by Hammerschmidt et al. demonstrated that C16:0 sphingolipids formed by CerS6 impair mitochondrial function in insulin-resistant liver of obese mice. In CerS6Δ/Δ mice, a significant reduction was observed in mitochondrial C16:0-Cer content (by approx. 60%). The rodents were also protected from the adverse metabolic effects of HFD-induced obesity and insulin resistance. Furthermore, it was observed that CerS6 ablation reduced C16:0 ceramide in MAM and MITO fractions (64). Interestingly, haploinsufficiency of CerS2 (the dominant form of hepatic CerS responsible for the synthesis of very long-chain ceramides C22:0/C24:0/C24:1) in heterozygous CerS2^-/+^ mice increased the susceptibility to diet-induced steatohepatitis by inhibition of mitochondrial β-oxidation, respiration and ATP production in hepatocytes (65). This was accompanied by a compensatory increase in C16:0-Cer, which further underlines the importance of different ceramide molecular species in the induction of mitochondrial dysfunction.

### Mitochondria-Associated Membranes and Mitochondrial Ceramide Transport

Mitochondria are spatially situated close to the endoplasmic reticulum – the site of *de novo* ceramide synthesis. Both organelles are closely associated and interact physically and functionally through mitochondria-associated membranes (66). MAM are responsible for the exchange of metabolites and calcium between ER and MITOS. Furthermore, they are enriched in enzymes participating in lipid synthesis and transport, and modulate cell signaling pathways involved in pathophysiological processes such as mitochondrial dysfunction and ER stress (67–71). The data indicate that sphingolipids synthesized mainly through a *de novo* pathway in ER may transfer to the mitochondria through MAM contact sites (72). MAM serve as potential sites of sphingolipid synthesis, mediate the exchange of lipids between membranes and regulate mitochondrial metabolic function, glucose homeostasis and insulin sensitivity (73). Indeed, Tubbs et al. demonstrated that a marked disruption of ER-mitochondria interactions is an early stage preceding mitochondrial dysfunction and insulin resistance in the myotubes of obese patients with or without T2D compared to healthy lean subjects. In addition, they demonstrated *in vivo* that defective ER-mitochondria coupling is closely linked to impaired muscle insulin sensitivity in mice and humans (74). Importantly, Theurey et al. reported that glucose and the pentose phosphate-protein phosphatase 2A (PP-PP2A) pathway regulate mitochondrial dynamics and function by controlling MAM integrity in the liver of diabetic mice. This result supports the role of MAM in the management of both glucose homeostasis and mitochondrial fitness associated with hepatic insulin resistance (75). The above data highlight a dual role of MAM-mitochondria interaction: as an early stage leading to mitochondrial dysfunction and an indirect role associated with the transport of sphingolipids from the ER to the mitochondria, which may result in mitochondrial dysfunction.

### CERT-Dependent Mitochondrial Ceramide

**Figure 2:**
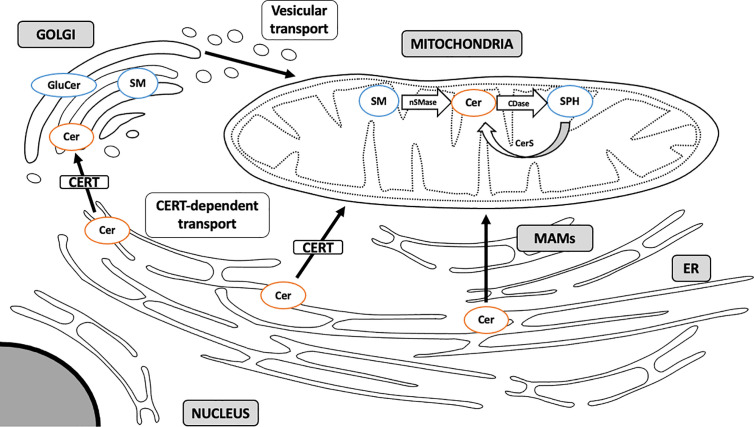
Sphingolipid transport to mitochondria. Sphingolipids are synthesized mainly through a *de novo* pathway located in ER. Complex sphingolipids are synthesized in Golgi membranes and travel to various intracellular compartments *via* vesicular transport. Ceramide can also translocate to the mitochondria directly through membrane contact sites at the MAM interface or through CERT-dependent mechanism. MAM, mitochondria-associated membranes; ER, endoplasmic reticulum; SM, sphingomyelin; Cer, ceramide; GluCer, glucosylceramide; SPH, sphingosine; CERT, ceramide transfer protein; nSMase, neutral sphingomyelinase; CDase, ceramidase; CerS, ceramide synthase. Arrows denote major routes of sphingolipid transport to mitochondria.

Due to a highly hydrophobic nature, ceramide is locked within cellular membranes and cannot diffuse intracellularly (76). ER ceramide synthesized *via* a *de novo* pathway is transported to the Golgi apparatus and transformed into complex sphingolipids, such as sphingomyelin and glucosylceramides (GluCer). This transport may occur *via* two mechanisms: by means of ceramide transfer protein (CERT), a non-vesicular ATP-dependent transporter, and through ATP-independent vesicular transport (59, 77) (**Figure 2**). Kumagai et al. showed that CERT efficiently transferred ceramides with C14:0, C16:0, C18:0, and C20:0 acyl chains (78). It has recently been demonstrated that CERT plays a pivotal role in controlling muscular ceramide content and insulin sensitivity. The study by Bandet et al. revealed that a decrease in CERT content induces ceramide accumulation in muscle cells through a faulty transport from the ER to the Golgi apparatus, which hinders SM biosynthesis. Inversely, increased CERT expression simultaneously intensifies sphingomyelin synthesis in Golgi membranes and increases insulin sensitivity in *in vitro* culture of skeletal muscle cells (79). Modulation of CERT compartmentalization can trigger ceramide-specific effect in the targeted organelle. Selective transport of ceramides by CERT to the mitochondria rather than the Golgi membranes induces apoptotic cell death. Jain et al. analyzed ceramide flow to mitochondria using mitoCERT (recombinant CERT protein equipped with the outer mitochondrial membrane anchor sequence) in HeLa cells and showed that its expression activates apoptosis and pro-apoptotic Bcl-2 protein Bax. These findings provide direct evidence that translocation of ER ceramides to the mitochondria through CERT protein triggers apoptotic cell death by a mitochondria-related mechanism (80). This suggests that some of the detrimental effects of mitochondrial ceramide accumulation can be governed by externally-supplied ceramide delivered through the CERT-dependent mechanism.

### Sphingomyelin as the Source of Mitochondrial Ceramide

In addition to ceramide, complex sphingolipids, such as sphingomyelin, can indirectly modulate insulin signaling, yet the overall SM effect is still under debate. SM is one of the major phospholipids of the plasma membrane and the most abundant sphingolipid in eukaryotic cells (81). Sphingomyelin synthase (SMS) is expressed in the form of two isoforms, SMS1 and SMS2. They catalyze sphingomyelin synthesis through the transfer of phosphocholine from phosphatidylcholine to the C-1 position of ceramide (50). Higher content of skeletal muscle ceramide and a lower level of sphingomyelin were observed in obese, glucose intolerant patients as compared to lean controls (82). *In vitro* ablation of SMS2 in C2C12 myotubes triggered ceramide accumulation and inhibition of insulin signaling. Although sphingomyelin levels were unchanged by these manipulations, SMS2-induced accumulation of ceramide negatively affected mitochondrial respiratory capacity and ATP synthesis (83). This points to ceramide rather than sphingomyelin as the relevant antagonist of insulin signaling and mitochondrial function in muscles, with sphingomyelin acting as a metabolic sink for bioactive ceramide and sphingoid backbones. In agreement with the above findings, it was confirmed that SMS2 knockout mice are resistant to diet-induced obesity and T2D (84, 85). The study by Mitsutake et al. revealed that SMS2 deficiency in mice prevents HFD-induced fatty acid uptake, lipid droplet formation and insulin resistance (84). The study by Huang et al. demonstrated that the inhibition of SMS2 activity increases IRS-1, Akt and GSK-3β phosphorylation in the liver of HFD-fed mice (85). However, it was also observed that sphingomyelin synthesis inhibits insulin signaling through c-Jun kinase (JNK) activation (86). These partly conflicting observations on the role of SM and SMS in insulin resistance can be explained by multifaceted effect of sphingomyelin which acts as both the buffer for excess ceramide and its source during sphingomyelin hydrolysis.

### Sphingolipid Salvage Pathway in Mitochondria

The catabolism of sphingolipids begins with the hydrolysis of sphingomyelin or glycosphingolipids by the action of various isoforms of sphingomyelinases and cerebrosidases, respectively. The above enzymes release ceramide which is degraded to free sphingoid base and fatty acid by 3 different ceramidase isoforms according to their pH optima: acid ceramidase (aCDase, ASAH1), neutral ceramidase (nCDase, ASAH2) and alkaline ceramidase (alCDase, ASAH3). Free sphingosine can be re-acylated to form ceramide by CerS or phosphorylated by sphingosine kinases to yield S1P. Sphingosine-1-phosphate can be dephosphorylated back to sphingosine by sphingosine phosphate phosphatase (SPP) or degraded by sphingosine-1-phosphate lyase (SPL) to phosphoethanolamine and hexadecanal, resulting in the breakdown of sphingoid backbone and its exclusion from sphingolipid metabolism (49) (**Figure 1**). This lysosomal salvage pathway recovers a free sphingoid base through the degradation of complex sphingolipids. Subsequently, the liberated sphingosine can be recycled to ceramide in an organelle with CerS activity (44). Although the salvage pathway involves mainly lysosomes and ER, it was demonstrated that SMase, CDase and CerS enzymes are transported to the mitochondria, which allows both the sphingosine salvage from SM and sphingosine re-esterification to Cer (36, 37). The mitochondrial localization of an independent sphingolipid salvage pathway may underline the importance of sphingolipid metabolism in the mitochondria.

## The Role of Sphingolipid Accumulation in Mitochondrial Dysfunction

**Figure 3:**
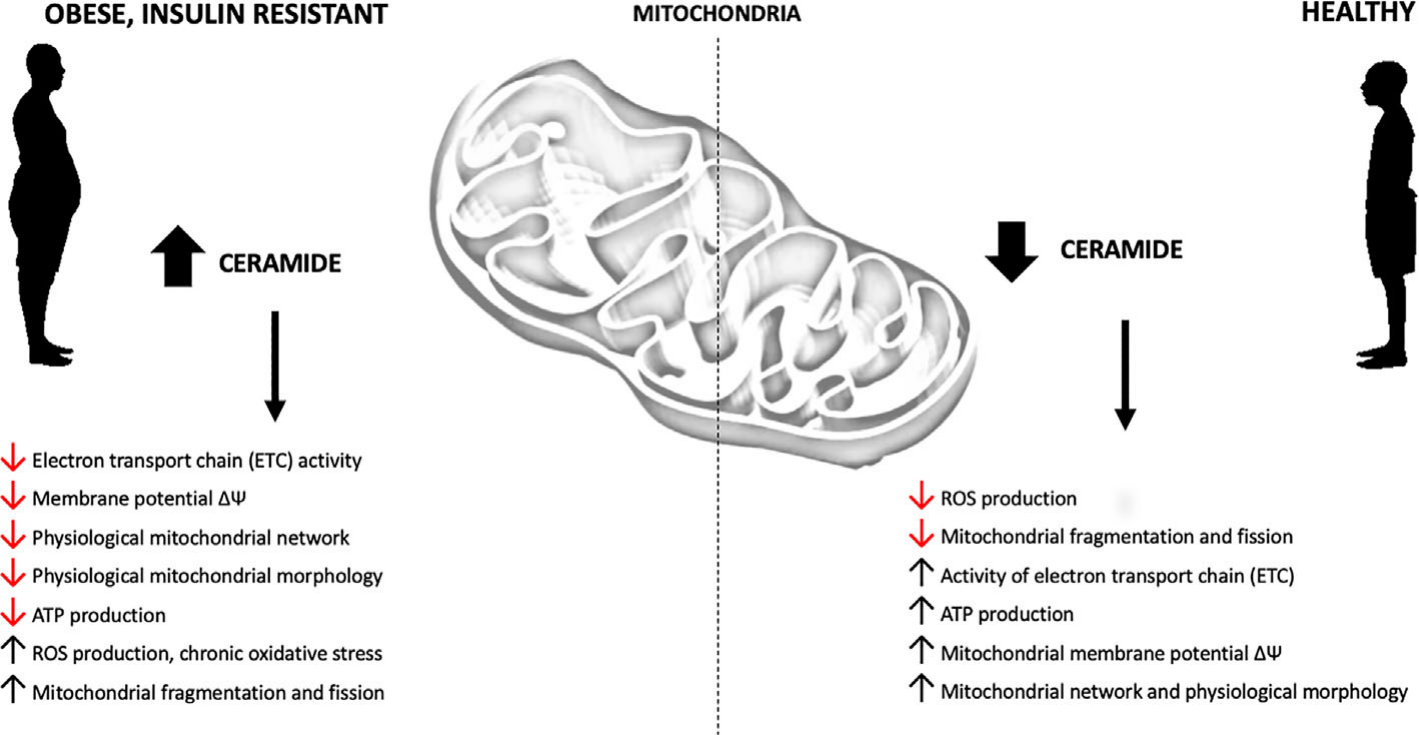
Obesity-related effects of ceramide accumulation on mitochondrial metabolism.

Recent studies, conducted on *in vivo* and *in vitro* models of obesity and T2D, support the conclusion that increased intracellular content of sphingolipids may impair mitochondrial function by interfering with the respiratory chain activity and other aspects related to mitochondrial bioenergetics (**Figure 3**, **Supplement Table 1**). Although this phenomenon is well established, the possible mechanisms are still under debate. Different studies have confirmed direct inhibition of mitochondrial respiration by ceramide, induction of mitochondrial fission and augmentation of ROS emission.

### Inhibition of Mitochondrial Respiration by Ceramide Accumulation

One of the hallmarks of mitochondrial dysfunction in T2D and obesity is decreased rate of mitochondrial respiration due to both direct and indirect impact of the above disorders on the mitochondrial respiratory chain function. This phenomenon has been well documented by several research groups. Disturbance of mitochondrial respiration was confirmed in the skeletal muscles of T2D subjects (87–89) as well as of obese individuals (88) as compared to lean volunteers. Moreover, detrimental effects were observed for almost every other aspect of mitochondrial metabolism directly connected with mitochondrial respiration, namely TCA cycle, membrane potential and OXPHOS (90). Interestingly, both in animal and human models it was demonstrated that negative effects of obesity on mitochondrial function could be ameliorated by the stimulation of muscle contraction, which both improves the mitochondrial respiratory function and corrects mitochondrial morphological changes (91, 92). In line with the above findings, it was observed that muscle contraction reduces global ceramide content, which improves insulin-stimulated glucose uptake (91). The link between ceramide accumulation and inhibition of mitochondrial respiration was further confirmed by *in vitro* experiments. The study by Di Paola et al. showed that both the short and long chain ceramides (namely C2:0-Cer and C16:0-Cer) inhibited the activity of ETC complex I and oxidation of NADH-linked substrates in isolated rat heart mitochondria (93). Similar findings were presented by Gudz et al. in rat heart mitochondria treated with C2:0-Cer with significantly inhibited respiratory chain activity (94). Another direct involvement of ceramide in the regulation of mitochondrial respiration was observed in an animal model of CerS6-ablated (CerS6^Δ/Δ^) mice, where ADP-stimulated complex III activity was significantly increased in isolated liver mitochondria from HFD-fed CerS6^Δ/Δ^ animals compared to rodents fed only HFD (64). Conversely, the study by Raichur et al. showed that CerS6 overexpression inhibits complex II of the electron transport chain, decreases Akt/PKB activation and promotes triglyceride accumulation in the liver, which points to C16:0-ceramide as key modulator of both the mitochondrial ETC chain activity and insulin sensitivity (65). The importance of C16:0 ceramide as a modulator of mitochondrial respiration was also confirmed in CerS2-null mice in which the ablation of very long-chain CerS2 triggered the compensatory accumulation of medium-chainC16:0 ceramide in isolated mitochondrial fractions and caused chronic oxidative stress, mitochondrial ROS emission and complex IV inhibition. These observations suggest that C16:0 ceramide, sphinganine and possibly sphingosine modulate the mitochondrial respiratory chain by direct inhibition of complex IV activity (95). Finally, human studies also revealed detrimental effects of mitochondrial ceramide accumulation. Perreault et al. observed a decrease in ADP-stimulated state 3 respiration under ceramide treatment (52). Interestingly, only C18:0 and C24:0 ceramides increased state 4 respiration, which suggests that those molecular species may enhance mitochondrial proton leak and reduce mitochondrial coupling between ATP synthesis and oxygen consumption.

Clues regarding the possible mechanisms of ceramide-mediated mitochondrial dysfunction arise from the studies on mitochondria-mediated apoptosis. Similar impairment of mitochondrial electron transport chain function was observed during the initiation of the apoptosis signaling cascade (96). Ceramide accumulation increased the permeability of outer mitochondrial membrane by assembling membrane ceramide channels and stimulated the release of pro-apoptotic intermembrane space proteins, including cytochrome c (Cyt c) (97–100). Cytochrome c plays numerous functions, including the transfer of electrons from complex III to complex IV, involvement in ROS production and mitochondrial protein import, and is a key regulator of apoptosis (101). Some of the adverse effects of mitochondrial ceramide accumulation in obesity and T2D could be explained by ceramide-mediated Cyt c efflux from the mitochondria. The release of cytochrome c is mediated by protein members of the B-cell lymphoma 2 (Bcl-2) family, such as Bak and Bax (102). Cytochrome c activates executioner caspases, thus concluding the process of programmed cell death (103). The assembly of ceramide channels in outer mitochondrial membrane is inhibited by anti-apoptotic Bcl-2/Bcl-xL and stimulated by the pro-apoptotic Bax protein (104, 105). Moreover, very long-chain ceramides synthesized by CerS2 disrupt mitochondrial membrane permeabilization, whereas medium-chain C16:0-Cer synthesized by CerS5 promotes membrane permeability and cytochrome C release (106). Interestingly, as already mentioned, heterozygous CerS2^-/+^ mice display increased susceptibility to diet-induced steatohepatitis, inhibition of mitochondrial β-oxidation, respiration and ATP production in liver hepatocytes (65). The studies quoted above suggest that medium-chain ceramide channel formation in the outer mitochondrial membrane is a plausible mechanism for ceramide-mediated inhibition of mitochondrial respiration due to Cyt c release. An alternative mechanism to ceramide channel formation is the direct interaction between Cer and voltage-dependent anion channel 2 (VDAC2), a mitochondrial platform for Bax/Bak translocation (107). VDAC opening creates a route for Cyt c release and decreases mitochondrial membrane potential (mitochondrial polarization) by disrupting the mitochondrial electron transport chain (108, 109). Finally, mitochondrial Cer is involved in the phosphorylation-mediated activation of p38 MAPK protein kinase, a key regulator of apoptosis in various cell types (110). The activation of p38 kinase is one of the constituents of ceramide-mediated apoptosis, leading to the disturbance of the mitochondrial transmembrane potential (111, 112). Although the above studies were performed to elucidate the role of ceramide in mitochondria-mediated apoptosis, it has been evidenced that similar molecular mechanisms could be responsible – at least partially – for the induction of mitochondrial dysfunction in insulin-resistant states. In the case of non-alcoholic fatty liver disease (NAFLD), ceramide-induced mitochondria-mediated hepatocyte apoptosis could be an important constituent of insulin resistance observed in non-alcoholic steatohepatitis (NASH). Both the early effects of ceramide accumulation, such as mitochondrial dysfunction and oxidative stress accompanied by the subsequent apoptosis of liver cells, are observed during the progression of NAFLD (113). Indeed, studies demonstrated that hepatic mitochondrial dysfunction precedes the development of NAFLD and insulin resistance in an obese rodent model (114). Similarly, in the liver of morbidly obese humans, simple steatosis displayed lower rate of hepatocyte apoptosis compared to cases of severe NASH (115). The link between hepatic Cer accumulation, mitochondria-mediated apoptosis and NAFLD was clearly displayed in the study of Jiang et al. where chronic inhibition of the *de novo* pathway of ceramide synthesis by myriocin (an SPT inhibitor) in HFD-fed NAFLD rats impaired both hepatic ceramide accumulation and apoptosis as well as decreased inflammation and steatosis and improved indices of insulin resistance (116). One of the recently discovered effects of ceramide accumulation on mitochondrial bioenergetics is the modulated expression of mitochondrial uncoupling proteins (UCPs) involved in thermogenesis. The study by Yang et al. demonstrated that the whole-body inhibition of *de novo* ceramide synthesis by myriocin in both the ob/ob and HFD-fed diabetic mice improves systemic oxygen consumption, increases UCP3 mRNA expression in epididymal fat pads and decreases both the whole-body adiposity and indices of insulin resistance at the systemic and tissue (skeletal muscle and liver) levels (117). The recent study by Chaurasia et al. elucidated the role of ceramides in the regulation of the metabolic activity of brown adipose tissue (BAT). Conditional, tissue-specific ablation of SPTLC2 gene (encoding catalytic subunit of SPT) in BAT decreased cellular Cer content, up-regulated UCP1 protein, increased energy expenditure and glucose homeostasis and prevented diet-induced obesity. Conversely, ablation of aCDase gene (encoding acidic ceramidase, ASAH1) led to ceramide accumulation in BAT, down-regulation of UCP1 protein, decline in energy expenditure, worsening of insulin resistance, and increase in fat accumulation (118). Modulation of BAT ceramide levels through SPTLC2 or aCDase ablation had the opposite effects on mitochondrial β-oxidation and morphology. Interestingly, the stimulation of BAT cells with isoproterenol (β-adrenergic receptor agonist) decreased the transcription of enzymes required for ceramide synthesis, which suggests that the said proceduremight be a possible universal mechanism of ceramide-mediated regulation of mitochondrial fitness. According to the recent data, dihydroceramide is unable to decrease the mitochondrial function, as opposed to bioactive ceramide (119–121). Ceramide desaturase 1 (Des1) ablation in ob/ob mice leads to the accumulation of sphinganine-based sphingolipid species (dihydrosphingolipids, missing double bond at C4-C5 position of sphingoid base) in both the liver and adipose tissue at the expense of sphingosine-based ceramide species. Animals lacking Des1 displayed improved indices of insulin sensitivity, decreased adiposity and increased oxygen consumption in white adipose tissue compared to ob/ob controls (122). The above study confirms that ceramide molecular species promote stronger biological activity than their dihydroceramide counterparts in relation to the alterations in mitochondrial function. Similar conclusions were reached regarding the involvement of Cer and dhCer in the direct inhibition of the insulin signaling pathway (123, 124) and in mitochondrial ceramide channel formation (125).

### Mitochondrial Morphological Changes in Obesity and Type 2 Diabetes

Kelly et al. analyzed skeletal muscle mitochondria in type 2 diabetic as well as obese subjects by electron microscopy and observed a decrease in size compared to the mitochondria of lean volunteers (89). Similarly, the study by Bach et al. showed that skeletal muscle mitochondrial network in obese Zucker rats was severely affected by fragmentation and led to a 25% decrease in mitochondrial volume as compared to lean controls (126). Furthermore, it was observed that the ablation of the proteins involved in maintaining mitochondrial dynamics – mitofusin 2 (MFN2) – caused alterations in mitochondrial morphology in both the liver and skeletal muscles (127). Hepatic mitochondrial morphology determined by transmission electron microscopy revealed a significantly reduced ratio between mitochondrial length and width in the hepatic mitochondria of obese mice, which suggested increased fragmentation of the mitochondrial network (64). The above data indicates that obesity considerably affects mitochondrial content, size and dynamics, suggesting that ectopic accumulation of lipids is a possible cause of these alterations. According to the recent findings, ceramide as a culprit of morphological changes in the mitochondrial network.

### Effect of Ceramide Accumulation on Mitochondrial Morphology, Fusion, Fission, and Mitochondrial Network Dynamics

Mitochondria are highly dynamic organelles which undergo fusion and fission, leading to the development of a vast range of mitochondrial forms, from singular separate organelles to a continuous, interconnected network (128). Mitochondrial network dynamics is regulated by processes of mitochondrial fusion, fission, biogenesis and mitophagy (129). Fission and fusion play critical roles in maintaining the functions of the mitochondria. Fusion generates healthy organelles by mixing the contents of partially damaged mitochondria with healthy ones, whereas fission is needed for expanding the mitochondrial network and complete removal of damaged mitochondria through mitophagy (130). Both processes were identified as features of either insulin resistance (fission) or proper insulin signaling (fusion) in cell-based and animal models of insulin resistance as well as in obese and/or T2D subjects (131). A growing amount of evidence suggests that the sphingolipid species balance and the expression of specific ceramide synthases play a crucial role in controlling mitochondrial dynamics (45).

The process of fission is regulated by the dynamin-like protein 1 (Drp1) and mitochondrial fission factor (Mff) protein which recruits Drp1 to the mitochondrial surface (132, 133). The study by Wang et al. demonstrated that liver-specific deletion of Drp1 in mice (Drp1LiKO) decreases the fragmentation of mitochondrial network, increases whole-body energy expenditure and hinders the development of diet-induced obesity and insulin resistance (134). Furthermore, it has been proven that the inhibition of fission-related proteins (Mff/Drp1/Fis1) enhances mitochondrial network and reverses IR by activating the IRS1-Akt pathway and GLUT4 translocation to the cell membrane, thus improving systemic insulin sensitivity, whereas the activation of fission by Drp1/Fis1 overexpression triggers the opposite effect (135). Regarding the impact of lipid overload on mitochondrial network dynamics, it was demonstrated that an excess of palmitate (PA) induces mitochondrial fragmentation and increases mitochondrion-associated Drp1 and Fis1 expression in differentiated C2C12 muscle cells. Mitochondrial network fragmentation is associated with mitochondrial depolarization, increased oxidative stress and reduced ATP production (136). The study by Smith et al. showed that palmitate or ceramide treatment in C2C12 myotubes induces mitochondrial fission through a Drp1-dependent mechanism (137). Ceramide increases Drp1 expression, whereas Drp1 inhibition resulting from Mdivi-1 treatment (inhibitor of Drp1) prevents ceramide-induced mitochondrial fission. Increased fission is associated with reduced mitochondrial respiration (at the level of complex II), increased ROS production and Akt/PKB inhibition. Ceramide-induced Drp1-mediated mitochondrial fission was also confirmed *in vivo* (137). Currently the strongest evidence for ceramide-specific control over the mitochondrial fission process comes from the study by Hammerschmidt et al. in which the ablation of CerS6 in a mouse model of HFD-induced insulin resistance facilitated a successful reversal of the fragmentation of hepatic mitochondrial network, rescued the insulin-sensitive phenotype and prevented HFD-induced obesity (64). The authors present convincing data to confirm that direct binding of CerS6-produced C16:0-Cer with Mff triggers Drp1 recruitment to the mitochondrial surface and stimulates mitochondrial fission that leads to insulin resistance.

Mitochondrial fusion combines mitochondria into a tubular interconnected network. The process is governed by two GTPases: mitofusin 1 (MFN1) and mitofusin 2 (MFN2), located in the outer mitochondrial membrane, and optic atrophy protein 1 (OPA1) in the inner mitochondrial membrane (138–140). It was observed that liver-specific ablation of fusion-promoting protein MFN2 in mice led to numerous metabolic abnormalities characterized by endoplasmic reticulum stress, increased ROS production, enhanced hepatic gluconeogenesis and impaired insulin signaling (127). Altered activity of MFN2 was reported in the skeletal muscles of both obese subjects and type 2 diabetic patients. Diminished expression of MFN2 is regarded as an important factor in the induction of mitochondrial dysfunction in the course of obesity and T2D (126). Similar effects of diminished MFN2 expression were observed in the liver and muscle cells in animal models of high-fat diet-induced obesity and insulin resistance, which supports the hypothesis that MFN2 can affect insulin signaling and insulin sensitivity through mitochondria-related mechanisms. MFN2 ablation in the mouse liver triggers mitochondrial dysfunction, thus enhancing ROS production and JNK activity as well as inactivating IRS1. Interestingly, these alterations were ameliorated by N-acetylcysteine (ROS scavenger) treatment (127).

### Oxidative Stress and ROS Production

Numerous studies indicated that obesity and type 2 diabetes have a major impact on redox balance (94, 141–143). The excess of ROS vs. the capacity of scavenging mechanisms increases oxidative stress, which results in oxidative damage to proteins, lipids, carbohydrates and nucleic acids. Excessive mitochondrial production of ROS may negatively affect the insulin signal transduction pathway and thus contribute to inhibition of insulin action and mitochondrial dysfunction (93, 144). Mitochondrial dysfunction and oxidative stress have been confirmed to be implicated in obesity and IR, but the underlying mechanisms are still unknown. Lefort et al. described decreased mitochondrial mass and high rates of ROS production in the mitochondria isolated from the skeletal muscles of obese insulin resistant individuals (145). Smith et al. demonstrated increased ROS levels and reduced Akt/PKB phosphorylation in C2C12 cell line treated with ceramides (137). Boosted ROS production induced by short-chain ceramides was also observed in rat heart and liver mitochondria (94, 144). Anderson et al. reported an increase of H_2_O_2_ in the skeletal muscles of both obese and insulin-resistant rodents and humans fed a high-fat diet, which suggests a correlation of mitochondrial H_2_O_2_ emission and the pathogenesis of IR (142). Zigdon et al. noted that C16 ceramide and/or sphinganine induce ROS formation by modulating the activity of mitochondrial complex IV, resulting in chronic oxidative stress in CerS2 null mice (95). Nonetheless, these findings suggest the possibility that mitochondrial ROS emission may be a key factor in the development of insulin resistance associated with high-fat diet and it is at least partially triggered by the accumulation of mitochondrial sphingolipids.

## Conclusions

A growing body of evidence connects sphingolipid accumulation with the abnormalities of mitochondrial metabolism detected in the insulin-sensitive tissues of obese or T2D subjects (**Supplement Table 1**). Most recent studies strongly support the hypothesis that the improvement of mitochondrial fitness through the modulation of specific ceramide species may counteract high-fat diet-induced insulin resistance. Adverse effects of ceramide accumulation on the mitochondria are most likely parallel to the direct influence of ceramide on the constituents of the signaling pathway.

- The metabolism of sphingolipids in different cellular compartments contribute to such pathological conditions as obesity, diabetes, insulin resistance and oxidative stress.
- A number of enzymes (ceramide synthase, ceramidase, sphingosine kinase, sphingomyelinase) involved in sphingolipid metabolism have been found within the mitochondria. This may indicate that at least partial independent sphingolipid metabolism occurs in the mitochondria.
- A significant amount of evidence has demonstrated that ceramides may regulate respiratory-chain components and consequently induce mitochondrial dysfunction at the level of ETC.
- Adverse changes in mitochondrial network dynamics are concomitant with the accumulation of intracellular sphingolipids. According to the recent studies, mitochondrial fission may depend on the interaction of the ceramide specific molecular species with key proteins responsible for the initiation of the fission process.
- Extensive research is required to investigate the organelle-specific role of sphingolipids, which will be especially beneficial for the identification of all ceramide-dependent metabolic abnormalities in the course of insulin resistance and type 2 diabetes.
- Focus of the future research on effective targeting of mitochondrial sphingolipids may lead to the development of new therapies for the treatment of both insulin resistance and mitochondrial dysfunction in T2D.

## Author Contributions

KR-O performed a literature search, drafted the manuscript, and co-authored the figures and tables. PZ drafted the manuscript, co-authored the figures and tables, and edited the final version of the work. All authors contributed to the article and approved the submitted version.

## Funding

The work was funded by the Medical University of Bialystok, grant No. SUB/1/1DIN/19/003/1117 and No. N/ST/ZB/18/003/1204.

## Conflict of Interest

The authors declare that the research was conducted in the absence of any commercial or financial relationships that could be construed as a potential conflict of interest.

## Abbreviations

3-KR, 3-ketosphinganine reductase; 3-KSph, 3-ketosphinganine; aCDase, acid ceramidase (ASAH1); ADP, adenosine diphosphate; Akt/PKB, serine/threonine kinase Akt (PKB); alCDase, alkaline ceramidase (ASAH3); ATP, adenosine triphosphate; BAT, brown adipose tissue; Bcl-2, B-cell lymphoma 2 protein; C1P, ceramide-1-phosphate; CDase, ceramidase; CEM, caveolin-enriched microdomains; Cer, ceramide; CERK, ceramide kinase; CerS, ceramide synthase; CERT, ceramide transfer protein; COX, cytochrome oxidase; Cyt c, cytochrome c; DES, dihydroceramide desaturase; Des1, ceramide desaturase 1; dhCer, dihydroceramide; dHSph, dihydrosphingosine (also described as sphinganine, Sph); Drp1, dynamin-related protein 1; Drp1LiKO, Drp1 liver-specific knockout mouse; ER, endoplasmic reticulum; ETC, electron transport chain; FA, fatty acids; GluCer, glucosylceramide; GLUT4, glucose transporter type-4; GM, ganglioside; HFD, high-fat diet; IR, insulin receptor; IRS-1, insulin receptor substrate 1; JNK, c-Jun kinase; KDSR, 3-ketodihydrosphingosine reductase; MAM –mitochondria-associated membranes; MCSs, membrane contact sites; Mff, mitochondrial fission factor; MFN1, mitofusin 1; MFN2, mitofusin 2; IMM, inner mitochondrial membrane; mitoCERT, recombinant CERT protein with mitochondria anchor sequence; OMM, outer mitochondrial membrane; mtDNA, mitochondrial DNA; NAFLD, non-alcoholic fatty liver disease; NASH, non-alcoholic steatohepatitis; nCDase, neutral ceramidase (ASAH2); nSMase, neutral sphingomyelinase; OPA1, optic atrophy protein 1; OXPHOS, oxidative phosphorylation; PA, palmitate; PHB2, prohibitin 2; PI3K, phosphatidylinositol 3-kinase; PIP2, phosphatidylinositol 4,5-bisphosphate; PIP3, phosphatidylinositol (3,4,5)-trisphosphate; PKB, protein kinase B; PKCζ, protein kinase C ζ (zeta); PP2A, protein phosphatase 2A; PP-PP2A, pentose phosphate-protein phosphatase 2A; ROS, reactive oxygen species; S1P, sphingosine-1-phosphate; SL, sphingolipids; SM, sphingomyelin; SMase, sphingomyelinase; SMS, sphingomyelin synthase; SPH, sphingosine; SphK, sphingosine kinase; SphK2, sphingosine kinase 2; SPL, sphingosine-1-phosphate lyase; SPP, sphingosine phosphate phosphatase; SPT, serine palmitoyl transferase; T2D, type 2 diabetes; TCA, tricarboxylic acid; UCPs, uncoupling proteins; VDAC2, voltage-dependent anion channel 2; βHAD, β-hydroxyacyl CoA dehydrogenase.

## Glossary

**Table:**

ACT	adoptive cell transfer
ADCs	adult stem cells
ADCC	antibody-dependent cellular cytotoxicity
AKT	activate protein kinase B
APCs	antigen-presenting cells
apCAFs	antigen-presenting CAFs
&alpha;SMA	alpha smooth muscle actin
CAFs	cancer-associated fibroblasts
CAR-T cells	chimeric antigen receptor T cells
CAV1	Caveolin 1
cCAFs	cycling CAFs
CD	cluster of differentiation
cDC	conventional dendritic cells
circRNAs	circular RNAs
COL1A1	collagen
type I	alpha 1
COL13A1	collagen type III alpha 1 chain
COL14A1	collagen type XIV alpha 1 chain
CCL5	chemokine (C-C motif) ligand 5
CSF	colony-stimulating factor
CTLA-4	cytotoxic T-lymphocyte-associated protein 4
CXCL12	C-X-C motif chemokine 12
cyTOF	time-of-flight mass spectrometry
DC	dendritic cell
dCAFs	developmental CAFs
DPP-4	dipeptidyl peptidase-4
EC	endothelial cell
ECM	extracellular matrix
EGF	epidermal growth factor
eNOS	endothelial nitric oxide synthase 3
EMTs	epithelial-mesenchymal transitions
ERK	extracellular-signal-regulated kinase
eRNAs	enhancer RNAs
ESCs	embryonic stem cells
ESCC	esophageal squamous cell carcinoma
FAP	fibroblast activated protein
FCGR3A	low affinity immunoglobulin gamma Fc region receptor III-A
FGF	fibroblast growth factors
FGFBP-2	fibroblast growth factor binding protein 2
FOXP3	forkhead box P3
GC	germinal center
HIF-1&alpha	hypoxia-inducible factor 1-alpha
HITT	
HIF-1&alpha	inhibitor at translation level
HLA	human leukocyte antigen
iCAFs	inflammatory CAFs
Ig	immunoglobulin
IGHG1	immunoglobulin heavy constant gamma 1
JNK	c-Jun N-terminal kinase
KLF2	Kruppel-like factor 2
KLRD1	Killer cell lectin-like receptor subfamily D, member 1
LRRC15	leucine-rich repeat containing 15
lincRNAs	large intergenic non-coding RNAs
ICB	immune checkpoint blockade
IFN	interferon
IL-6	interleukin6
IL-11	interleukin 11
IL-1&beta	interleukin 1 beta
LAG3	lymphocyte-activation gene 3
LN	lymph nodes
lncRNAs	long non-coding RNAs
mAb	monoclonal antibody
MALAT1	metastasis-associated lung adenocarcinoma transcript 1
MAPK	mitogen-activated protein kinase
mCAFs	matrix CAFs
MHCII	major histocompatibility complex class II
miRNAs	MicroRNAs
MMP9	matrix metallopeptidase 9
MMTV-PyMT	mouse mammary tumor virus-polyoma middle tumor-antigen
Morrbid	myeloid RNA repressor of BCL2L11 induced death
MSCs	mesenchymal stem cells
mTOR	mammalian target of rapamycin
NCRs	natural cytotoxicity receptors
NEAT1	noncoding nuclear-enriched abundant transcript 1
NF-&kappa;B	nuclear factor kappa-light-chain-enhancer of activated B cells
NK	natural killer
NKG2D	natural killer group 2D
NOX4	NADPH oxidase 4
PDAC	pancreatic ductal adenocarcinoma
pDC	plasmacytoid DCs
PDGF	platelet-derived growth factor
PD-L1	programmed death-ligand 1
piRNAs	Piwi-interacting RNAs
PRC2	Polycomb repressor complex 2
ROS	reactive oxygen species
S100A4	S100 Calcium Binding Protein A4
scRNAseq	Single-cell RNA sequencing
SIGLEC1	sialic acid binding Ig like lectin 1
siRNAs	small interfering RNAs
SLAM	signaling lymphocyte activation molecule
sno-lincRNAs	Intron-derived small nucleolar lincRNAs
SOX-4	SRY (sex determining region Y)-box 4
STAT3	Signal transducer and activator of transcription 3
STEEL	spliced-transcript endothelial-enriched lncRNA
TAMs	tumor-associated macrophages
TCR	T cell receptor
TECs	tumor endothelial cells
TGF-&beta1	transforming growth factor beta 1
TIGIT	T cell immunoreceptor with Ig and ITIM domains
TIM-3	T-cell immunoglobulin and mucin-domain containing-3
TME	tumor microenvironment
TNF-&alpha	tumor necrosis factor alpha
TP53	Tumor protein 53
TRAIL	TNF-related apoptosis-inducing ligand
Treg	regulatory T cells
UTR	untranslated region
VEGF	vascular endothelial growth factor
vCAFs	vascular CAFs
vWF	von Willebrand factor
Wnt	Wingless-related integration site
YB-1	Y-box binding protein
ZEB1	Zinc Finger E-Box Binding Homeobox 1
