# Supplementary material for: Sphingolipids as a Culprit of Mitochondrial Dysfunction in Insulin Resistance and Type 2 Diabetes
Source: Front Endocrinol (Lausanne). 2021 Mar 18;12:635175. doi: 10.3389/fendo.2021.635175 (PMC8013882; doi:10.3389/fendo.2021.635175)
Supplement: Supplementary file 1 [file Table_1.docx]

| **Tissue** | **Model** | **Alternations in mitochondrial metabolism** | **Affected lipid species** | **Enzymes involved** | **Changes in insulin resistance** | **Reference** |
| --- | --- | --- | --- | --- | --- | --- |
| Skeletal muscle (human biopsy) | T2D  OBESITY | - Decreased activity of electron transport chain proteins - TCA, OXPHOS - Mitochondrial membrane potential | ND | ND | Impaired insulin signaling | Mogensen et al. [1] |
| Heart tissue  (rat) | N-acetyl sphingosine(C2:0-Cer)  stimulation | - Inhibition of electron transport chain (ETC) complex I activity | ND | ND | ND | Di Paola et al. [2] |
| Heart tissue  (rat) | N-acetyl sphingosine (C2-Cer) stimulation | - Decreased activity of respiratory chain complex III - Increased ROS production | ND | ND | ND | Gudz et al. [3] |
| Skeletal muscle (human biopsy) | T2D  OBESITY | - Decreased activity of NADH-O_2_ oxidoreductase - Reduced mitochondrial content - Fragmented, small mitochondria | ND | ND | Impaired insulin signaling | Kelley et al. [4] |
| Skeletal muscle (human biopsy) | T2D  OBESITY | - Decreased activity of respiratory chain complex I - Decreased activity of NADH-O_2_ oxidoreductase | ND | ND | Impaired insulin signaling | Ritov et al. [5] |
| Skeletal muscles (human biopsy)  isolated rat mitochondria | C18:0- and C24:0- Cer stimulation | - Decrease in ADP-stimulated activity of respiratory chain complex III - Increase in oxidative stress | ND | ND | ND | Perreault et al. [6] |
| Skeletal muscles | T2D | - Mitochondrial phosphorylation (reduced by 30%) | ND | ND | Impaired insulin signaling, decrease in insulin sensitivity | Petersen et al. [7] |
| Hepatocytes  (mice) | Wild-type HFD-fed mice | - Decrease in ADP-stimulated activity of respiratory chain complex III - Mitochondrial fission, impairment of mitochondrial function | ↑ C16:0- Cer  ↓ C16:0- Cer | ND | Impaired insulin signaling, decrease in insulin sensitivity (HFD-fed mice) | Hammerschmidt et al. [8] |
|  | HFD-fed CerS6Δ/Δ mice | - ADP-stimulated complex (III) activity significantly increased in HFD-fed CerS6Δ/Δ mice - Mitochondrial fission, improvement of mitochondrial function |  | Ablation of CerS6 | Improved systemic insulin sensitivity in HFD-fed CerS6Δ/Δ mice |  |
| Skeletal muscles  (mice) | HFD-fed mice, inhibition of CerS1 | - Inhibition of mitochondrial fatty acid oxidation - Increase in the respiratory activity of complex I, II and IV accompanied by increased activity of TCA cycle enzyme citrate synthase and increasedβ-oxidation - Inhibition of mitochondrial fission | ↓ C18:0- Cer | Inhibition of CerS1 | ND | Turner et al. [9] |
| Hepatocytes  (CerS2 null mice) | CerS2 null mice | - Direct inhibition of respiratory chain complex IV - ROS formation, chronic oxidative stress | ↑ C16:0-Cer  ↑ sphinganine | Ablation of CerS2 | ND | Zigdon et al. [10] |
| Primary hepatocytes (C57BL6/J mice) | HFD-fed mice, CerS6 overexpression | - Inhibition of Akt/PKB phosphorylation - Inhibition of electron transport chain complex II | ↑ C16:0-Cer | Overexpression of CerS6 | Impaired insulin signaling, decrease in insulin sensitivity | Raichur et al. [11] |
| Skeletal muscles (Zucker rats) | HFD-fed mice | - Decreased expression of MFN2   (by 34%) vs lean controls   - Modified mitochondrial network vs. lean controls (25% fragmentation) - Depression of OXPHOS - Impairment of mitochondrial fusion | ND | Ablation of MFN2 | ND | Bach et al. [12] |
| Hepatocytes  (MFN2 null mice) | HFD-fed Mfn2 ablation mice | - Alterations in mitochondrial morphology - Enhanced hepatic gluconeogenesis   ↑ ROS production | ND | Ablation of MFN2 | Impaired insulin signaling, decrease in insulin sensitivity | Sebastián et al. [13] |
| C2C12 muscle cells  (mice) | N-acetyl sphingosine (C2-Cer) stimulation | - Increased mitochondrial fission - Increased production of ROS - Reduced Akt/PKB phosphorylation - Reduced mitochondrial respiration (complex II activity) | ND | ND | ND | Smith et al. [14] |
| C2C12 muscle cells  (mice) | palmitate treatment | - Induced mitochondrial fragmentation, increased fission - Increased oxidative stress - Loss of ATP production - Mitochondrial depolarization | ND | Inhibition of Drp1 | Reduced insulin-stimulated glucose uptake | Jheng et al. [15] |

[1] M. Mogensen, K. Sahlin, M. Fernström, D. Glintborg, B.F. Vind, H. Beck-Nielsen, and K. Højlund, Mitochondrial respiration is decreased in skeletal muscle of patients with type 2 diabetes. Diabetes 56 (2007) 1592-9.

[2] M. Di Paola, T. Cocco, and M. Lorusso, Ceramide interaction with the respiratory chain of heart mitochondria. Biochemistry 39 (2000) 6660-8.

[3] T.I. Gudz, K.Y. Tserng, and C.L. Hoppel, Direct inhibition of mitochondrial respiratory chain complex III by cell-permeable ceramide. J Biol Chem 272 (1997) 24154-8.

[4] D.E. Kelley, J. He, E.V. Menshikova, and V.B. Ritov, Dysfunction of mitochondria in human skeletal muscle in type 2 diabetes. Diabetes 51 (2002) 2944-50.

[5] V.B. Ritov, E.V. Menshikova, J. He, R.E. Ferrell, B.H. Goodpaster, and D.E. Kelley, Deficiency of subsarcolemmal mitochondria in obesity and type 2 diabetes. Diabetes 54 (2005) 8-14.

[6] L. Perreault, S.A. Newsom, A. Strauss, A. Kerege, D.E. Kahn, K.A. Harrison, J.K. Snell-Bergeon, T. Nemkov, A. D'Alessandro, M.R. Jackman, P.S. MacLean, and B.C. Bergman, Intracellular localization of diacylglycerols and sphingolipids influences insulin sensitivity and mitochondrial function in human skeletal muscle. Jci Insight 3 (2018).

[7] K.F. Petersen, S. Dufour, D. Befroy, R. Garcia, and G.I. Shulman, Impaired mitochondrial activity in the insulin-resistant offspring of patients with type 2 diabetes. The New England journal of medicine 350 (2004) 664-71.

[8] P. Hammerschmidt, D. Ostkotte, H. Nolte, M.J. Gerl, A. Jais, H.L. Brunner, H.G. Sprenger, M. Awazawa, H.T. Nicholls, S.M. Turpin-Nolan, T. Langer, M. Krüger, B. Brügger, and J.C. Brüning, CerS6-Derived Sphingolipids Interact with Mff and Promote Mitochondrial Fragmentation in Obesity. Cell 177 (2019) 1536-1552.e23.

[9] N. Turner, X.Y. Lim, H.D. Toop, B. Osborne, A.E. Brandon, E.N. Taylor, C.E. Fiveash, H. Govindaraju, J.D. Teo, H.P. McEwen, T.A. Couttas, S.M. Butler, A. Das, G.M. Kowalski, C.R. Bruce, K.L. Hoehn, T. Fath, C. Schmitz-Peiffer, G.J. Cooney, M.K. Montgomery, J.C. Morris, and A.S. Don, A selective inhibitor of ceramide synthase 1 reveals a novel role in fat metabolism. Nat Commun 9 (2018) 3165.

[10] H. Zigdon, A. Kogot-Levin, J.W. Park, R. Goldschmidt, S. Kelly, A.H. Merrill, Jr., A. Scherz, Y. Pewzner-Jung, A. Saada, and A.H. Futerman, Ablation of ceramide synthase 2 causes chronic oxidative stress due to disruption of the mitochondrial respiratory chain. J Biol Chem 288 (2013) 4947-56.

[11] S. Raichur, S.T. Wang, P.W. Chan, Y. Li, J. Ching, B. Chaurasia, S. Dogra, M.K. Öhman, K. Takeda, S. Sugii, Y. Pewzner-Jung, A.H. Futerman, and S.A. Summers, CerS2 haploinsufficiency inhibits β-oxidation and confers susceptibility to diet-induced steatohepatitis and insulin resistance. Cell Metab 20 (2014) 687-95.

[12] D. Bach, S. Pich, F.X. Soriano, N. Vega, B. Baumgartner, J. Oriola, J.R. Daugaard, J. Lloberas, M. Camps, J.R. Zierath, R. Rabasa-Lhoret, H. Wallberg-Henriksson, M. Laville, M. Palacín, H. Vidal, F. Rivera, M. Brand, and A. Zorzano, Mitofusin-2 determines mitochondrial network architecture and mitochondrial metabolism. A novel regulatory mechanism altered in obesity. J Biol Chem 278 (2003) 17190-7.

[13] D. Sebastián, M.I. Hernández-Alvarez, J. Segalés, E. Sorianello, J.P. Muñoz, D. Sala, A. Waget, M. Liesa, J.C. Paz, P. Gopalacharyulu, M. Orešič, S. Pich, R. Burcelin, M. Palacín, and A. Zorzano, Mitofusin 2 (Mfn2) links mitochondrial and endoplasmic reticulum function with insulin signaling and is essential for normal glucose homeostasis. Proc Natl Acad Sci U S A 109 (2012) 5523-8.

[14] M.E. Smith, T.S. Tippetts, E.S. Brassfield, B.J. Tucker, A. Ockey, A.C. Swensen, T.S. Anthonymuthu, T.D. Washburn, D.A. Kane, J.T. Prince, and B.T. Bikman, Mitochondrial fission mediates ceramide-induced metabolic disruption in skeletal muscle. Biochem J 456 (2013) 427-39.

[15] H.F. Jheng, P.J. Tsai, S.M. Guo, L.H. Kuo, C.S. Chang, I.J. Su, C.R. Chang, and Y.S. Tsai, Mitochondrial fission contributes to mitochondrial dysfunction and insulin resistance in skeletal muscle. Mol Cell Biol 32 (2012) 309-19.
